# Fungal biomass and ectomycorrhizal community assessment of phosphorus responsive *Pinus taeda* plantations

**DOI:** 10.3389/ffunb.2024.1401427

**Published:** 2024-05-28

**Authors:** Jacob Hackman, Alex Woodley, David Carter, Brian Strahm, Collin Averill, Rytas Vilgalys, Kevin Garcia, Rachel Cook

**Affiliations:** Department of Forestry and Environmental Resources, North Carolina State University, Raleigh, NC, United States

**Keywords:** phosphorus, ectomycorrhiza, *Pinus taeda*, biomass, microbiome, rhizosphere, mesh bags

## Abstract

Ectomycorrhizal fungi and non-ectomycorrhizal fungi are responsive to changes in environmental and nutrient availabilities. Although many species of ectomycorrhizas are known to enhance the uptake of phosphorus and other nutrients for *Pinus taeda*, it is not understood how to optimize these communities to have tangible effects on plantation silviculture and P use efficiency. The first step of this process is the identification of native fungi present in the system that are associated with *P. taeda* and influence P uptake efficiency. We used sand-filled mesh bags baited with finely ground apatite to sample ectomycorrhizal and non-ectomycorrhizal fungi associated with the rhizosphere of P-responsive *P. taeda* under several field conditions. Mesh bags were assessed for biomass accumulation over three years using a single three-month burial period pre-harvest and three six-month burial periods post-planting. Amplicon sequencing assessed ectomycorrhizal and non-ectomycorrhizal communities between phosphorus treatments, sites, mesh bags, and the rhizosphere of actively growing *P. taeda* in the field. We found biomass accumulation within the mesh bags was inversely related to increasing phosphorus fertilization (carryover) rates from pre-harvest to post-planting. Up to 25% increases in total biomass within the bags were observed for bags baited with P. Taxonomic richness was highest in Alfisol soils treated with phosphorus from the previous rotation and lowest in the Spodosol regardless of phosphorus treatment.

## Introduction

Ectomycorrhizal fungi communities respond to changes in environmental conditions and nutrient availability (Smith and Read, 2008). Pine plantations in the southeastern U.S. are often deficient in bioavailable nutrients, particularly phosphorus (P), due to the inherent P deficiency of the highly weathered and acidic soil groups that dominate the region (Comerford et al., 2002). These soils are hypothesized to be in a “terminal steady state” of P depletion as the parent material slowly loses its P content (Walker and Syers, 1976). As a result of this deficiency, loblolly pine (*Pinus taeda* L.) responds well to P fertilization, which can increase their overall productivity (Albaugh et al., 2019). Although P fertilization has been used on these sites since the late 1960s, it is now reserved for sites that will benefit most in terms of crop tree growth response due, in part, to the rising cost of fertilizer.

Additionally, research emerged, finding that for some highly weathered soils, a single application of P fertilizer was enough to supply an entire stand for the entirety of a 20-year rotation (Pritchett and Comerford, 1982; Turner and Lambert, 2002). Considering the conservative and cyclical nature of forest systems in recycling nutrients back into the soil from organic material (Kiser and Fox, 2012), additional research was proposed that some residual P would remain to supply subsequent rotations as carryover P. This carryover P was found to be a significant source of P into subsequent rotations, potentially supplying a site with three or more years into a forest stand rotation (Everett and Palm–Leis, 2009). This P was also found to be sensitive to the magnitude of the application of P from the previous rotation; however, the most optimal method to determine carryover P rates early in a stand rotation remains challenging to determine and appears to vary by site characteristics (Hackman et al., 2024). Finding additional methods to track carryover P from previous rotations will assist growers in making accurate management decisions to improve overall yield and soil health from one rotation to the next.

With and without P additions, trees rely on symbiotic ectomycorrhizal fungi (ECM) to enhance their ability to absorb P from the soil. These fungi colonize the cortex of pine roots and create a network of hyphae that trade soil minerals for carbohydrates from the plant (Doidy et al., 2012). The tree provides the ECM with carbohydrates in the form of sugars, and the ECM transfers water and nutrients from the soil to colonized roots via specific transport proteins (Garcia et al., 2016; Becquer et al., 2019). ECM fungi can increase the nutrient assimilation capacity of P by up to two orders of magnitude by increasing the total root surface area (Smith and Read, 2008) and assimilating non-available P by releasing acid phosphatases and siderophores from external hyphae (Ho, 1989; Cummings and Weinstein, 1990; Courty et al., 2010; Plassard and Dell, 2010).

The availability of P in soils directly affects the growth of ECM fungi, their nutrient exchange, and their interactions with host plants. Research has consistently shown that P levels in plants are much higher when they form associations with ECM fungi than those without (Smith and Read, 2008). However, under extreme P-limiting conditions, more carbon is allocated to ECM hyphal exploration into the soil to scavenge for nutrients at the expense of above-ground host biomass allocation (Bahr et al., 2015). P fertilizer additions in nutrient-limited environments have been linked to significant effects on the biomass and community composition of the ECM fungal communities. P additions in P-limited environments are associated with reduced underground biomass for roots and ECM fungi, favoring communities of low-biomass taxa specialized for short-distance soil exploration (Treseder, 2004; Bae et al., 2015; Bahr et al., 2015). In contrast, high-biomass ECM species specialized for long-distance soil exploration thrive in P-limited environments (Wallander and Nylund, 1992). In summary, hyphae production increases under deficient P levels, and when P is sufficiently available, fungal biomass and hyphal exploration are suppressed (Jones et al., 1990; Wallander and Nylund, 1992; Callahan et al., 2016).

We hypothesize ECM fungi have the potential to be used as a biological indicator for P availability in loblolly pine plantations in the southeastern U.S. Fine mesh bags, which exclude all plant roots but allow fungal hyphae to enter, can be “baited” with a P source to evaluate the community of ECM fungi (Hagerberg et al., 2003; Hedh et al., 2008; Callahan et al., 2016). Preliminary analyses conducted by Tacilla Villanueva (2015) on our two sites indicate that increasing rates of P fertilization led to increased productivity responses of *P. taeda* in each location. Because of this increase in productivity associated with increased rates of P fertilization, and thus P carryover, we assume the ECM community will shift to communities adapted to higher levels of P in the soil. By connecting productivity responses with the characterization of fungal communities and biomass, it may be possible to predict responses to fertilization better, as ECM fungi are crucial to nutrient uptake for *P. taeda* and are sensitive to nutrient inputs.

The primary purpose and goal of this experiment was to evaluate how legacy P fertilizations from a previous rotation affect fungal biomass and fungal communities between two distinct sites. To achieve this goal, a total of five objectives were created: (i) evaluate fungal biomass accumulation across four burial periods in two distinct sites; (ii) determine the primary drivers of biomass accumulation differences between sites and P carryover treatments; and (iii) determine if biomass is related to increased fungal diversity between carryover treatments and sites. (iv) Determine which treatments drive the community composition of ECM and non-ECM taxa extracted from mesh bags and rhizosphere, and (v) determine if the ECM or non-ECM communities in the mesh bags are associated with the rhizospheres of *P. taeda.*

## Methods

### Site description and design

This study builds on a long-term experiment established by the Forest Productivity Cooperative (formerly Forest Nutrition Cooperative, Albaugh et al., 2015) to investigate the effects of N and P fertilization rates on *P. taeda* productivity. The two sites chosen for this experiment are located on the Southeastern Atlantic Coastal Plain. Site selection was performed based on the proximity of sites, harvest schedule, and distinct soil characteristics of each site. The first site in northeastern Florida is a poorly-drained, fine, mixed, active, thermic, typic Albaqualf (Meggett series, CRIFF group A) with marine sediment parent material. The second site, located in southeast Georgia, is a somewhat poorly drained, sandy over loamy, siliceous, active, thermic Typic Haplohumods (Leon series, CRIFF group D), with marine sediment parent material, multiple spodic horizons, and no argillic or kandic horizon present within the first 100 cm of soil depth (NRCS, 2010) (**Table 1**).

**Table 1:** First rotation and second rotation site designs and descriptions.

Site	County and State	Latitude	Longitude	Species	Study Establishment	“Base” Site Index*	Years Since P Fertilization	Harvest Date
**Alfisol**	Nassau, Florida	30.6661 N	81.8361 W	Loblolly	1999	45	2004	Jan-19
**Spodosol**	Brantley, Georgia	31.3353 N	81.8217 W	Loblolly	1998	67	2005	Jan-19

*Base Site Index at 25 years old.

The first rotation of the experiment was harvested in 2019 and will be referred to as the “first rotation.” The current rotation is referred to as the “second rotation.” Experimental treatments are arranged at each site in a completely randomized block design with two replicates. A two-row buffer of trees surrounds each measurement plot. Each treatment plot contains six rows with 12 trees per row for approximately 70 trees per treatment, all labeled with aluminum tags to track individual tree growth and mortality over time. The first rotation plots received various cumulative N + P fertilizer applications at different rates and frequencies over six years. Treatments for the second rotation were overlaid on previous rates and either received an additional re-fertilization of P (40 + 45 kg P ha^-1^) applied at tree establishment as broadcast triple super phosphate or no fertilizer P (X + Y, where X = the P carryover rate and Y = re-fertilization rates) at the establishment (**Table 2**). The second rotation P treatment labels for the remainder of this publication are (in kg ha^-1^): 0 + 0 P, 40 + 0 P, 40 + 45 P, and 121 + 0 P. All plots, except the control, received N as urea plus a urease inhibitor, K as KCl, and a micronutrient mix to remove nutrient limitations besides P (**Table 2**). All treatments received 0.177 L of imazapyr as a herbicide after bedding in the spring for herbaceous weed control.

**Table 2:** P carryover treatments include carryover rates from the first rotation (established in 1999) and re-carryover rates from the second rotation (established in 2019).

	First Rotation		Second Rotation		
P Carryover Treatments	Cumulative P	Cumulative N	N	K	P
	*kg ha^-1^*				
*0 + 0 P* *40 + 0 P* ***40 + 45 P*** *121 + 0 P*	0 40 40 121	0 400 400 1210	52 52 52 52	29 29 29 29	0 0 **85** 0

Only 40 + 45 P carryover treatments received an additional 45 kg P ha^-1^ at planting the second rotation. Bold treatment received fertilization at the start of the second rotation.

### Mesh bag construction and deployment

**Figure 1:**
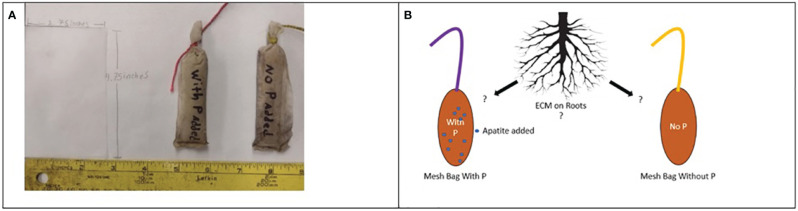
Single sheets of 50 um nylon mesh were cut out and folded to create a bag, then sealed via melting **(A)**. Bags were individually labeled and equipped with two colors of nylon twine to assist in relocating and differentiating between treated and untreated bags. **(B)** Hypothesis: ECM on the roots scavenging for P will accumulate in bags baited with P and not in bags without P.

Mesh bags were constructed using a 50 μm nylon mesh (9 x 5 cm), which is small enough to exclude fine plant roots but not fungal hyphae (Wallander et al., 2001), and filled with 30 grams (g) of acid-washed fine quartz sand sieved to 2 mm. In total, one hundred 128 bags were constructed for each field location; half of the bags in each site received a treatment of 1 gram of finely ground apatite (0–18-0) added to each bag and homogenized (P-treated). The remaining half were left as control bags filled only with sand (P-untreated). Each treatment plot received 16 bags (eight P-treated and eight untreated). Our working hypothesis for this additional P is that the P-treated bags will incentivize colonization of ECM and other fungi in these P-deficient environments. These bags were evenly buried between the bed and interbed throughout the plot. Each bag was buried 15 cm below the soil surface using a soil auger. Bags burials were marked using flagging and colored nylon string tied to the tops of each bag affixed to the flagging (**Figure 1**). Previous sequencing analyses using similar mesh bags found ECM fungi dominate the community compositions within the bags on mature tree stands (Parrent and Vilgalys, 2007; Wallander et al., 2013). Considering our two sites were recently harvested and established, causing a significant amount of disturbance, we expect the saprotrophic load of the fungi in the bags to be much higher than in previous studies.

Four burial periods were used in this experiment: one three-month burial period pre-harvest in 2018 and three six-month burial periods post-planting occurring consecutively from the spring of 2020 to the winter of 2022. After each six-month burial period, bags were harvested from the soil and separated by site, location, and mesh bag treatment. Mesh bag samples collected from the field after each burial period were stored on ice and frozen at -20°C (**Table 3**). To homogenize samples based on site and fertilizer treatment, mesh bags were opened and finely ground using a mortar and pestle. This grinding procedure ensured an even distribution of biological material in the sample. Subsequently, samples were split in half; one half was stored in the freezer for DNA extraction, while the other half was subjected to loss-on-ignition treatment. Although the loss-on-ignition method is initially intended for soil organic carbon quantification (Tiessen et al., 1993), we assume the fungal hyphae will combust similarly.

**Table 3:** The sampling timeline for fungal biomass and DNA sampling.

Burial period	2018 Pre-Harvest (-3–0 Months)	2020 Post (0–6 months)	2021 Post (6–12 months)	2022 Post (12–18 months)
**Stand Period**	Pre-harvest	Post-planting	Post-planting	Post-planting
**Fungal Biomass**	Collected	Collected	Collected	Collected
**DNA**	Not-collected	Collected	Not-collected	Not-Collected

The 2018 Burial period was three months, and 2020, 2021, and 2022 were installed consecutively for 6-month burial periods. DNA was only collected for 2020 post-planting.

While the method cannot distinguish how much biomass is ECM vs. non-ECM (Martin et al., 1990), we propose loss-on-ignition as a viable method to assess fungal biomass increases or decreases when using the mesh bag capture method. After covering samples with foil, samples were dried in an oven for 12 hours at 40°C, subsampled using 20 ml ceramic crucibles, and labeled with a wax pen. Ceramic crucibles were pre-heated to 100°C to eliminate residual moisture. Samples were weighed using a high-accuracy scale to the nearest milligram and inserted into a blast furnace heated to about 650°C for 12 hours to burn any hyphae present. The difference in weight between the initial and final measurements was used to estimate the amount of organic material and hyphae that accumulated in the bags over the burial period interval.

### Rhizosphere sampling and DNA extraction

We harvested fine roots from five dominant *P. taeda* trees in each treatment plot to collect root sections and stored them at -20°C for downstream use. The root clippings were placed in a 50 ml centrifuge tube with ten large glass beads and vortexed for 2 minutes with distilled H_2_O. We then extracted the root clippings from the centrifuge tube and vortexed the remaining fluid for 3 minutes at 10,000 x g. We subsampled 0.25 g for DNA extraction from the remaining pellet using DNeasy Powersoil Pro kits from QIAGEN. The mesh bags were independently grouped by burial period, site, and treatment.

Substrates from the mesh bags were also sampled for DNA. A total of 30 g of substrate was collected from each site and treatment; these samples were then finely ground using a mortar and pestle. Samples were diluted using 30 ml of clean distilled H_2_O as an eluent and ten large glass beads. After vortexing for 1 minute to suspend any remaining biomass in the supernatant, we extracted the supernatant from the sample using a 10 ml pipette after the majority of the sand and other heavy minerals settled, which was then centrifuged at 10,000 x g for 3 minutes. The supernatant was drained, and 0.25 g of the pellet was collected for further downstream DNA extraction using the DNeasy Powersoil Pro DNA extraction kit from QIAGEN.

The DNA quality and quantity for the rhizosphere and mesh bag samples were checked using a Thermo Scientific™ NanoDrop™ One Microvolume UV-Vis Spectrophotometer and standardized to approximately 10 ng mg^-1^. The rhizosphere and mesh bag samples had fungi targeted using the fungal standard ITS1f (Gardes and Bruns, 1993) and ITS4 (White et al., 1990) primers to amplify the fungal ITS region by PCR. The samples were then sequenced using the Illumina Miseq v3 (2 × 300 bp for fungi) platform at the North Carolina State University Genomic Sciences Laboratory. All remaining rhizosphere and mesh bag samples are stored at -20°C, awaiting additional potential for further downstream analysis (DNA extraction and metagenomic analysis).

### Statistics

Biomass samples were analyzed using a combination of mixed model designs and generalized linear regressions in JMP (JMP Pro v16.0; SAS Institute Inc., Cary, NC) to explore interactions between burial periods, mesh bag treatments, and P carryover treatment effects. The first mixed model design contained three fixed effects between the burial period, mesh bag treatment, and carryover treatment blocked by the site, with the location of the sample collected treated as a random effect. Variable importance was assessed from this mixed model using independent uniform random inputs to determine which factors contributed to biomass variation in the mesh bags. This method estimates variability based on the range of variation caused by each factor, independent of the model type and fitting method. To determine how biomass changed by burial period, biomass samples were blocked by site, mesh bag treatment, and the fixed effect treatment was burial period. Carryover treatment was the fixed effect on biomass between sites and within each burial period to assess P carryover treatment effects. The location was treated as random and was blocked by burial period, mesh bag treatment, and site. To determine whether biomass was related to increasing carryover rates, generalized linear regressions were constructed between carryover rates and biomass blocked by site, burial period, and mesh bag treatments. Multiple comparisons were performed on the mean total biomass accumulated from each burial period and separated using Tukey’s HSD with an alpha level of 0.05. Alpha diversities between samples were blocked by site and burial period and analyzed by P carryover treatment using one-way ANOVA on Shannon diversity indices with singletons removed generated using R v.4.0.0. Normalized community data was analyzed by site, mesh bag treatment, P carryover treatments, and location separated by communities within the mesh bags or on the rhizosphere and were generated by one and two-way PERMANOVA analysis using the statistical software package PAST ver3.0 (Hammer and Harper, 2001).

### Illumina sequence processing and analysis

**Figure 2:**
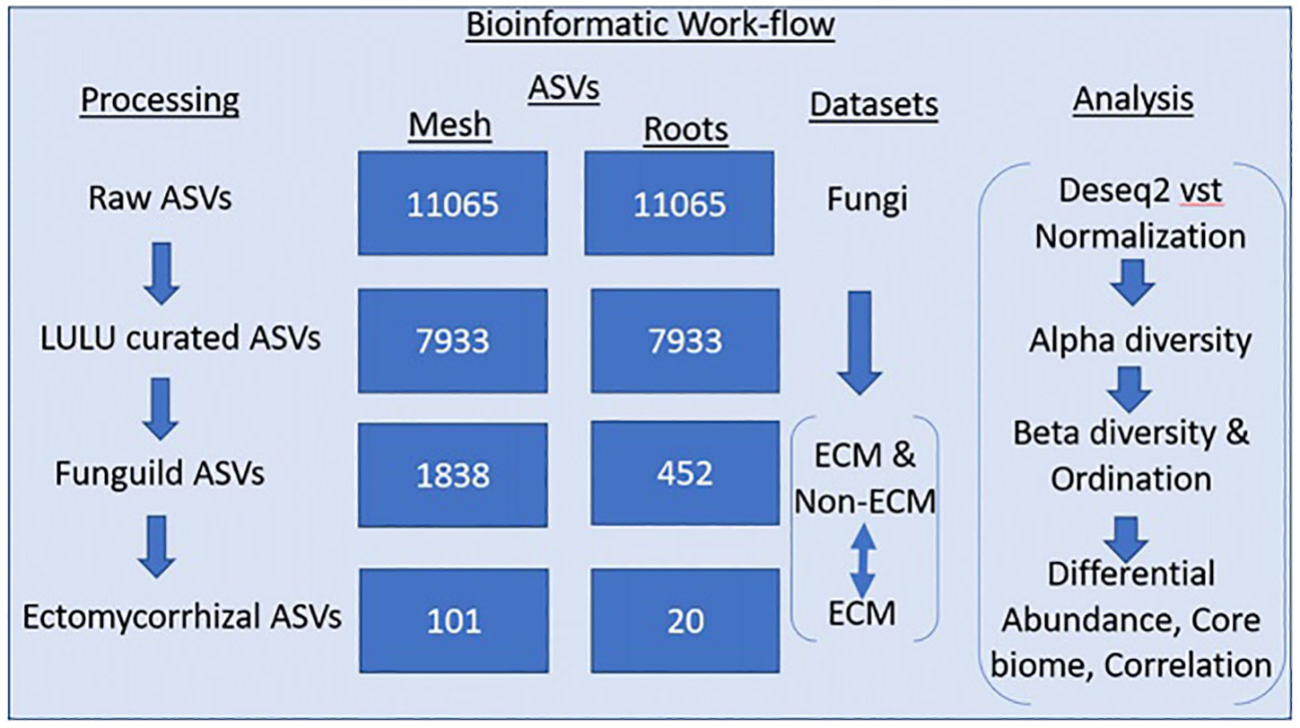
The processing column shows the removal of erroneous amplicon sequence variants (ASVs) performed using LULU, and the functional assignment was performed using Funguild. Two Funguild datasets were created: an ECM and non-ECM dataset and an ECM-only dataset. An individual analysis of each dataset can be found in the analysis column.

Illumina data was processed with dada2 v 1.10.6 (Callahan et al., 2016) in R v 4.2.2. Default parameters in the dada2 pipeline were used to create amplicon sequence variants (ASVs). Sequences were trimmed, and low-quality ends were removed. After filtering and merging, 11,605 fungal ASVs remained in the dataset, comprising 7,145,573 reads. After chimera removal, 734 ASVs were removed for a dataset with 11,065 fungal samples. Taxonomy for the dataset was assigned using the Unite vers. 9.0. fungal database for ITS reads. For improved estimates of biodiversity within the dataset, a post-clustering curation algorithm called LULU (Frøslev et al., 2017) was used to remove erroneous ASVs. After LULU curation, 3132 ASVs were removed, leaving 7933 ASVs. Functional parsing of ASVs into saprotrophic and mycorrhizal fungi guilds was performed using the FunGuild functional database (Nguyen et al., 2016). Of the remaining 7933 ASVs, FunGuild assigned 1966 ASVs to functional guilds (**Figure 2**).

### Bioinformatic analysis and processing of FunGuild datasets

FunGuild assigned taxa were subset into four datasets normalized separately using variance stabilizing transformation in the DeSeq2 package in R (Love et al., 2014). The four datasets were (1) mesh bag ECM, (2) rhizosphere ECM, (3) mesh bag non-ECM (4) rhizosphere non-ECM. Rhizosphere samples were analyzed by site and P carryover treatment, while mesh bags were analyzed by site, P carryover treatment, and mesh bag treatment. To determine what factors influence the composition of fungal sequences within each sample, Bray-Curtis dissimilarity-based permutation analysis of variance was performed for each treatment group for mesh bags and rhizosphere bootstrapped 9999 permutations to calculate a 95% confidence interval PASTv3 (Hammer and Harper, 2001). Alpha diversity measures were calculated using each treatment group’s effective number of species. This value is found by first finding the Shannon diversity index (H) and taking the exp of that value (Jost, 2006). The top 3 phyla and genera were calculated using Fantaxtic (Teunisse, 2022) on the relative abundances of sequences in all four datasets. Principal coordinate analyses (PCoAs) were constructed using DESeq normalized data and Bray-Curtis distances. Differential abundances of sequences between treatment groups were determined and calculated using the DESeq2 package in R. All datasets were analyzed using a 0.05 alpha value.

## Results

### Biomass changes by burial period and site

**Figure 3:**
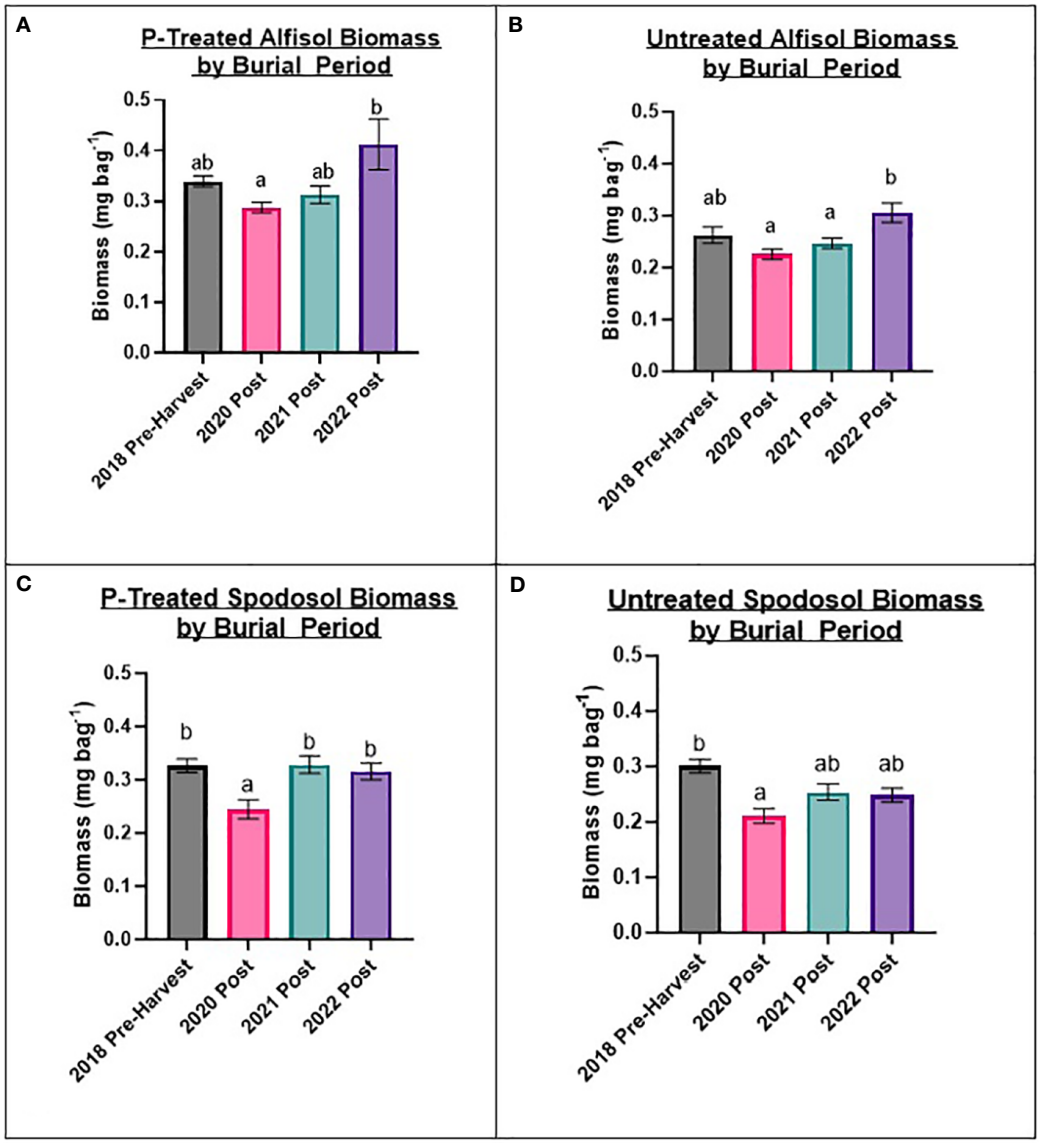
Results from biomass by burial period for mesh bag treatments by Alfisol and Spodosol. Biomass accumulations were higher in bags treated with P than in P-untreated bags. The lowest biomass accumulation occurred in the 2020 post-planting set of bags for most treatments and sites. Error bars represent standard error from the burial period mean. Letters represent Tukey’s HSD significant differences between burial periods using a 0.05 alpha value. **(A)** P-treated Alfisol Biomass; **(B)** P-untreated Alfisol Biomass; **(C)** P-treated Spodosol Biomass **(D)** P-untreated Spodosol Biomass.

To evaluate factors influencing fungal biomass, variables were determined from the most impactful to least impactful for changes in biomass for each site: burial period, mesh bag treatment, and P carryover treatments. This does not imply that P carryover had no effect, but P carryover contributed the least to the overall variation between sample biomass and was dependent on burial period, mesh bag treatment, and site. Biomass was strongly influenced (p-value ≤ 0.01) by the burial period for the mesh bags for each site. For the Alfisol and Spodosol, samples collected in 2020 post, just after the harvest, had the lowest overall accumulation of biomass across all burial periods. Additionally, the pre-harvest 2018 soil samples accumulated significantly more biomass than the 2020 post-burial period for the Spodosol, and trends could be observed for the Alfisol with similar results (**Figure 3**). The fact that pre-harvest bags buried for only three months accumulated as much, if not more, biomass than bags from subsequent burial periods that lasted 90 days longer suggests that biomass accumulation in these bags is probably not a linear response over time. Post-harvest burial periods, starting with 2020, indicate that biomass accumulation in the bags increased in the subsequent burial periods. Within each burial period, biomass was higher in P-treated mesh bags than in untreated mesh bags across all treatments (p-value ≤ 0.01), resulting in a 29.6% increase in total biomass in bags in P-treated bags vs. P-untreated bags for the Alfisol and a 19.36% increase for the Spodosol (**Figure 3**).

### Primary drivers of biomass accumulation

To determine the primary drivers of biomass accumulation, a generalized linear regression model constructed for each site showed that biomass responses to P carryover rates fluctuated depending on burial period, P treatment, and site (**Table 4**). Only the P-treated bags on the Alfisol in the last burial period in 2022 had a positive relationship to increases in the P carryover rate. In contrast, Spodosol biomass was negatively correlated with P carryover in the untreated 2021 P-treated 2018 and positively correlated in the untreated 2020 and P-treated 2020. The Spodosol and the Alfisol had similar trends in response to P carryover treatments. In the 2018 P-treated bag samples, the control treatment (0 + 0 P) had the highest biomass. Conversely, in the 2020 samples, we saw the opposite trend, where P-treated bags had greater biomass in higher P carryover application rates (40 + 45 P and 121 + 0 P) (**Supplementary Figure 1**).

**Table 4:** Generalized linear regression results from fungal biomass response to increases in P carryover rate by the site (Alfisol & Spodosol), bag treatment (C & T), and burial period.

Site	Treat	Burial period	DF	SS	F	R^2^	P	Eq.
***Alfisol***	P-untreated	2018	1	0.00664	1.35	0.070	0.26	y=0.2351 + 0.0005x
		2020	1	0.00133	0.66	0.035	0.43	y=0.2132 + 0.000224x
		2021	1	0.00162	0.81	0.045	0.38	y=0.2328 + 0.0000249x
		2022	1	0.00016	0.02	0.001	0.89	y=0.3099–0.0000771x
	P-treated	2018	1	0.00346	1.68	0.090	0.21	y = 0.3598–0.000364x
		2020	1	0.00344	1.84	0.097	0.19	y=0.2677 + 0.000363x
		2021	1	0.00040	0.07	0.004	0.80	y=0.3063 + 0.000123x
		2022	1	0.19985	5.16	0.233	0.04*	y=0.2584 + 0.0002763x
***Spodosol***	P-untreated	2018	1	0.00348	1.36	0.089	0.26	y=0.3267–0.000042x
		2020	1	0.01046	4.78	0.255	0.05*	y=0.1657 + 0.0000728x
		2021	1	0.01770	5.58	0.259	0.03*	y=0.2986–0.0000817x
		2022	1	0.00060	0.22	0.015	0.65	y=0.2378 + 0.000174x
	P-treated	2018	1	0.01145	5.13	0.255	0.04*	y=0.3624–0.000657x
		2020	1	0.02509	5.31	0.249	0.03*	y=0.1911 + 0.000972x
		2021	1	0.00094	0.20	0.014	0.66	y=0.3418–0.0000218x
		2022	1	0.00379	0.85	0.050	0.37	y=0.2951 + 0.000378x

The alpha value was 0.05, with significant relationships marked with a (*).

### Fungal diversity increases with biomass are site-dependent

**Figure 4:**
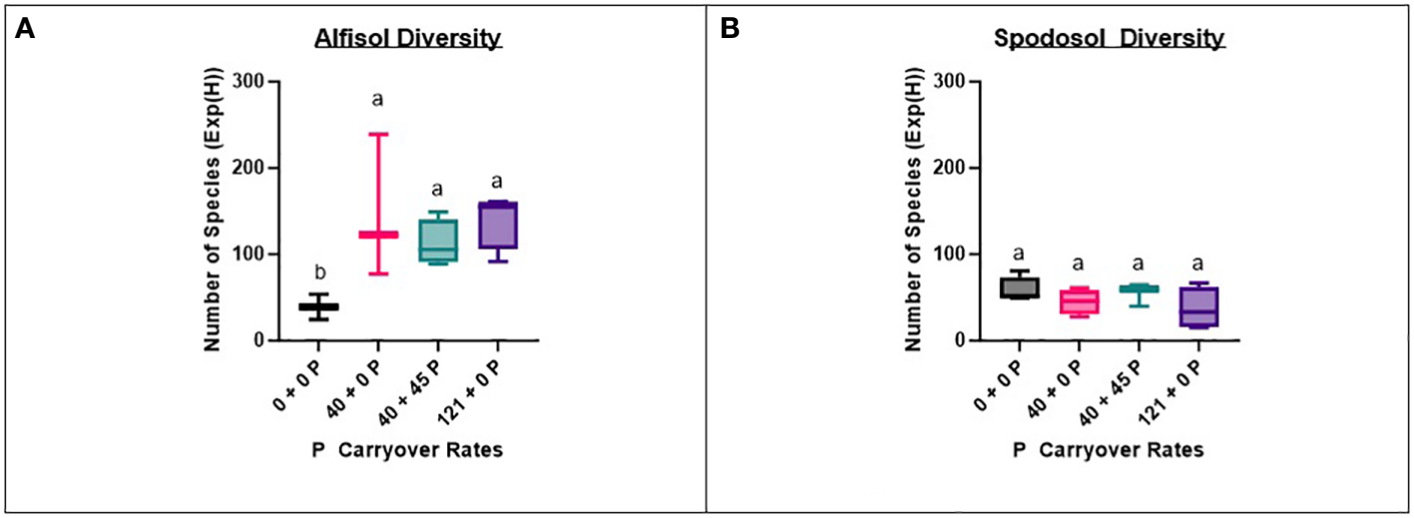
Fungi dataset species diversities for P by site. Overall, the Spodosol **(B)** had less effective species [Exp(H)] than the Alfisol **(A)** across P carryover treatments except for the control (0 + 0 P), which had a similar number of species as the Alfisol. The Alfisol had more fungal species in carryover treatments than the control (0 + 0 P). The number of species is obtained using the Shannon diversity index (H) exponent.

To determine if fungal community diversity was related to P carryover treatments, the number of taxa (Jost, 2006) was determined by P carryover treatment on the un-trimmed fungi dataset with singletons removed. Only P-treated bags located on the Alfisol had a strong positive relationship between increasing the P carryover treatment rate and increasing number of taxa (p-value = 0.0149). Results of this interaction revealed that any P carryover treatment on the Alfisol had, on average, a 212% increase in diversity compared to control treatments that did not receive any additional P carryover (**Figure 4**).

To determine if biomass in the bags was related to the number of species detected within those bags, we compared the biomass results directly to the effective number of species determined using the exponent of the Shannon-weaver index (Jost, 2006). We found the control treatment in the Alfisol (0 + 0 P) and the highest P carryover treatment in the Spodosol (121 + 0 P) had moderate relationships (R^2^: 0.657, p-value = 0.0147; R^2^: 0.591, p-value = 0.0258), respectively, to total biomass accumulated in those treatments (**Supplementary Figure 2**). Results of this interaction show that P-treated bags on the Alfisol had a significantly higher number of species and their total abundances than P-treated bags on the Spodosol.

### FunGuild taxonomic assignments

The FunGuild functional assignment resulted in a total of 1966 taxa combined from the rhizosphere and mesh bags after singleton removal. These 1966 taxa were placed into five groups 1: non-mycorrhizal (1,773 taxa), ectomycorrhizal (110 taxa), arbuscular mycorrhizal (71 taxa), ericoid mycorrhizal (5 taxa) and ectomycorrhizal – ericoid mycorrhizal (7 taxa). Of the 1966 taxa, only 193 were characterized as mycorrhizal fungi, of which 20 were detected on the rhizosphere and 182 were detected within the mesh bags. Of the 182 mycorrhizal fungi in the mesh bags, 55% were classified as ectomycorrhizal fungi (Parrent and Vilgalys, 2007; Wallander et al., 2013). Samples collected on the rhizosphere were dominated by ectomycorrhizal fungi, with over 80% belonging to ectomycorrhizal taxa. Of the 71 arbuscular mycorrhizas in the mesh bags, only *Acaulospora*, an arbuscular mycorrhiza genus, was found in the rhizosphere samples, which we assume was colonizing nearby herbaceous vegetation. Of the 110 ECM taxa detected, 101 belonged to the ECM mesh bag dataset, and 20 were in the rhizosphere ECM dataset.

### Drivers of community differences between ECM and non-ECM datasets

**Figure 5:**
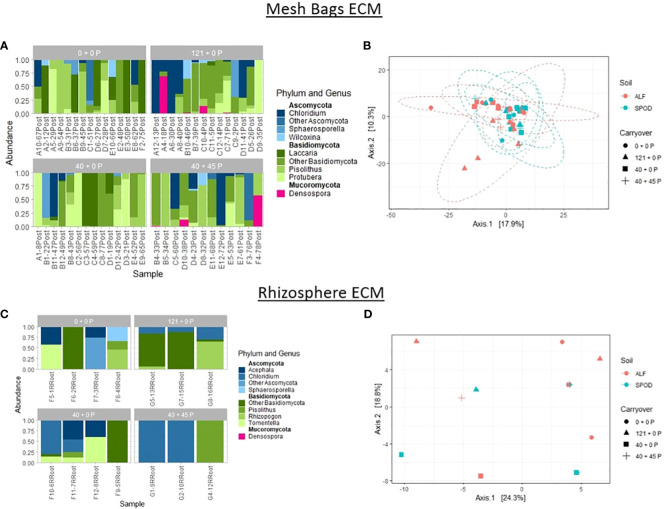
Relative abundance of fungal sequences illustrated using principal coordinate analyses (PCoA) graphs of mesh bags and rhizosphere ECM-only data. Principal coordinate analyses were constructed using DESeq2 normalized data projected using Bray-Curtis differences. Ellipses were constructed using a 95% standard deviation around carryover treatments. **(B)** Mesh bag PCoA of differences between P carryover treatments and site. **(C)** Rhizosphere relative abundances of top three most abundant phyla and genera. **(D)** PCoA of differences between P carryover treatments and site.

PERMANOVA analyses and PCoA graphs were generated to determine and visualize changes to the community composition for the site, carryover, and mesh bag treatments without making assumptions about the distribution. The betadisper function was used in the vegan package to test for overdispersion. One-way PERMANOVA analysis revealed strong effects on community diversity for the site (p-value ≤ 0.01) and no effect for carryover (p-value = 0.113) as the primary drivers of diversity for the ECM dataset. Samples collected from the rhizosphere for the ECM dataset were not affected by site or carryover. Testing between site and P carryover treatments revealed no effect for P carryover (p-value = 0.08) and a substantial effect for the site (p-value < 0.01) but no interaction effect. The top three most abundant phyla in the ECM samples were Basidiomycota, Ascomycota, and Mucoromycota. The top three most abundant genera from Ascomycota in the mesh bags were *Sphaerosporella, and Wilcoxina*, and from Basidiomycota were *Laccaria, Pisolithus*, and *Protubera* (Ford et al., 1985). Finally, the last phylum, Mucoromycota, only had a single enigmatic genus, *Denospora* (He et al., 2023). Rhizosphere samples were much less diverse, and the top six most abundant sequences collected from the Ascomycota and Basiciomycota were *Acephala, Sphaerosporella, Pisolithus, Rhizopogon*, and *Tomentella* (Molina and Trappe, 1994) (**Figure 5**).

**Figure 6:**
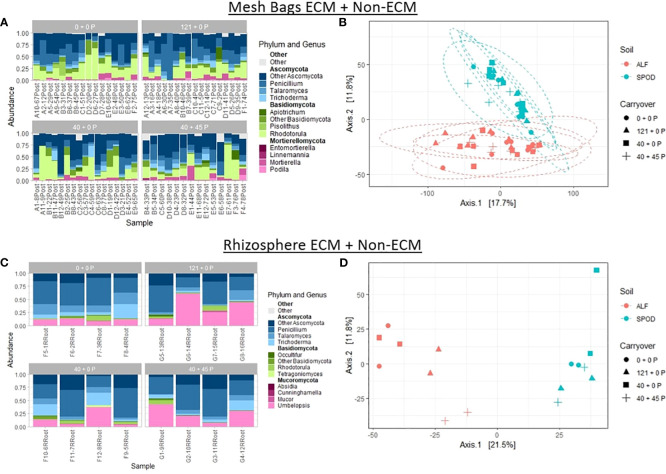
Relative abundances and principal coordinate analyses (PCoA) graphs of mesh bags and rhizosphere ECM + non-ECM data. Principal coordinate analyses were constructed using DESeq2 normalized data projected using Bray-Curtis differences. Ellipses were constructed using a 95% standard deviation around carryover treatments. **(A)** Mesh bag relative abundances of top three most abundant phyla and genera. **(B)** Mesh bag differences between P carryover treatments and site. **(C)** Rhizosphere relative abundances of top three most abundant phyla and genera. **(D)** PCoA of differences between P carryover treatments and site.

For the non-ECM dataset, one-way PERMANOVA analysis revealed a powerful effect for the site (p-value ≤ 0.01) and no effect for carryover treatments (p-value = 0.12) for P-treated mesh bags. For rhizosphere samples, the site had a strong effect on community composition (p-value ≤ 0.01). Two-way PERMANOVA analysis did not reveal significant interactions between treatments for either mesh bag or rhizosphere samples. Two-way PERMANOVA analysis revealed strong and moderate effects for the site (p-value < 0.01) and carryover (p-value = 0.04) (**Figure 6**).

### Differential abundances of taxa between sites

**Figure 7:**
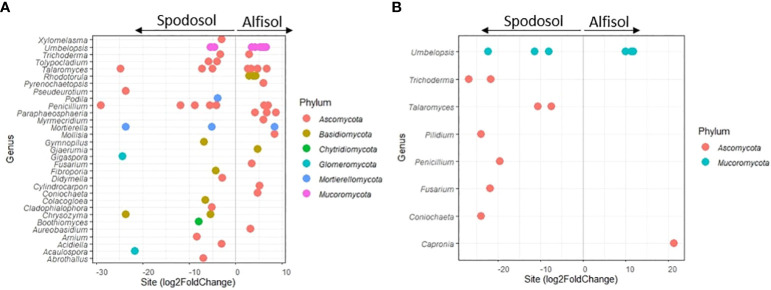
**(A)** non-ECM mesh bags and **(B)** non-ECM rhizosphere. Differential abundance of sequences by sites. Any dot indicates a significant log2FoldChange in genus level abundance (adjusted p-value < 0.05). log2FoldChange indicates the fold change in the number of counts found in that site, i.e., a log2fold change of positive two means there was a 4-fold increase in the number of sequences for those taxa for the Alfisol. Genera sequence abundances were normalized via DESeq2. The log2foldchange indicates the difference in fungal sequence abundance in sites.

Differential abundance analysis was performed using the DESeq2 package in R between sites and carryover treatments on the ECM and non-ECM datasets to determine if community abundances and distributions of taxa were unique to any individual treatment. For the non-ECM dataset, 55 genera were differentially abundant between sites within the mesh bags and 14 genera in the rhizosphere (**Figure 7**; **Supplementary Table 2**). For the ECM dataset, only *Denospora* was differentially abundant between the control treatment and the high treatment 121 + 0 P for the mesh bags, and no taxa were detected for the rhizosphere treatments. The most abundant taxa in the mesh bags and on the rhizosphere for the non-ECM dataset (base mean > 100) consisted of Ascomycota, while on the rhizosphere, more Mucoromycota were found. None of the 101 ECMs were found to be differentially abundant between sites.

### Shared taxa between treatments and datasets

**Figure 8:**
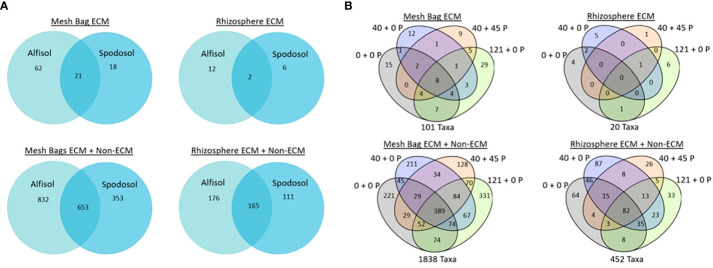
Potential common core of shared taxa between sites **(A)** site and **(B)** carryover rates were separated by mesh bag, and the two datasets separated rhizosphere.

**Figure 9:**
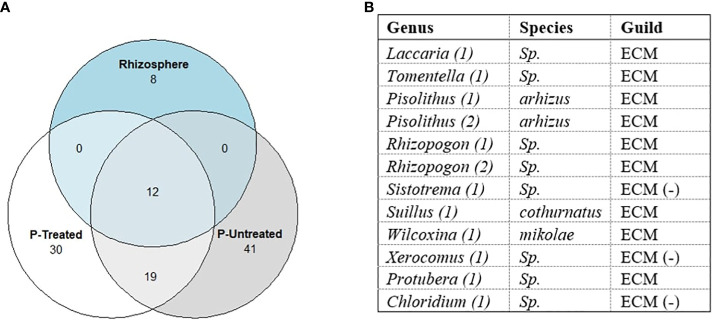
**(A)** Potential common core of raw ECM dataset sample counts show the 110 taxa classified as Ectomycorrhiza distribution between P-treated, P-untreated, and rhizosphere samples. Only 12 sequences were found in the rhizosphere and mesh bags, some of which were assigned the same taxa (1,2). **(B)** Rhizosphere and mesh bag shared taxa. Twelve taxa were found to be shared between the rhizosphere and the mesh bags treated with P. Only three Amplicon Sequence Variant (ASVs) were classified to the species level. Samples marked with (-) are possible ECM species but are not known as early colonizers of P. taeda.

One of our objectives was to determine if ECM taxa in the mesh bags were also known colonizers of *P. taeda* in the field. Special attention was given to this common core between the rhizosphere, P-treated, and P-untreated mesh bags (**Figure 8**). We wanted to determine whether mesh bags and rhizosphere share common taxa between taxa; 20 ECM taxa were detected from the rhizospheres of *P. taeda* trees. Of these taxa, 12 were unique to the Alfisol, six were unique to the Spodosol, and only two rhizosphere taxa were shared between the two sites, a *Chloridium* sp. and *Acephala macrosclerotiorum*. For mesh bag samples, 62 were unique to the Alfisol, 18 for the Spodosol, and 21 were shared between the two sites. The rhizosphere ECM + non-ECM dataset had 452 taxa found on the rhizosphere and 1838 taxa in the mesh bags across sites. Of the 1838 total taxa in the mesh bags, 832 were only on the Alfisols, 353 were on the Spodosol, and 653 were shared. Of the 452 taxa on the rhizosphere, 176 were unique to the Alfisol, and 111 were unique to the Spodosol (**Figure 8**). Of the 110 ECM taxa, 12 were shared between mesh bag treatments and the rhizosphere (**Figure 9**). A total of 19 taxa were unique to the mesh bags regardless of treatment. Eight were only found within the rhizosphere.

Of the twelve taxa shared between the rhizosphere and P-treated mesh bags, all genera found except *Sistotrema*, *Chloridium*, and *Xerocomus* are known early successional colonizers of *P. taeda* in North Carolina (Hackman et al., 2022). BLAST results of the sequences from the three genera against the UNITE database (Nilsson et al., 2019) could not discern any further details on possible species. These genera could be artifacts generated by the FunGuild database, and many species in these genera are not traditionally ECM (**Figure 9**). PERMANOVA and differential abundance analysis of mesh bag treatment revealed no significant differences between ECM and non-ECM datasets (p-value = 0.53, p-value = 0.69, respectively). Differential abundance analysis between mesh bags and rhizosphere revealed that members of *Protubera* and *Pisolithus* were more strongly associated with mesh bags than with the rhizosphere. Additional unique ASVs by treatment can be found in the **Supplementary Information** section (**Supplementary Table 1**).

## Discussion

This is the first study to observe fungal biomass and community responses to P carryover in *P. taeda* forest systems. This research is an integral first step in determining how soil type and P availability influence fungal biomass and fungal community composition in early rotation *P. taeda* plantations. The responses to site and P carryover treatments indicate that the ECM community and biomass are highly responsive to changes in environment and nutrient availabilities. We tracked changes to biomass across four burial periods for each site to determine how and if fungal biomass is affected by site, carryover P rates, and time. The burial period dramatically influenced biomass accumulation within the mesh bags. Samples collected in 2018 had the additional benefit of having large and established organic and litter layers where most ECM biomass is typically located in mature forest systems (Harvey et al., 1976). Samples collected in 2020, six months after site preparation and planting, accumulated less biomass than pre-harvest samples in 2018. We attribute this shift in biomass to harvesting and site preparation, which cause significant disturbances to standing biomass and the forest floor. In our case, harvesting was performed via a clear-cut, and thus, all standing biomass was removed from the plots. Site preparation on this site was bedded, which implies an extensive turnover of forest floor and integration of organic material into the subsoil.

Additionally, this site preparation technique also removes all living above-ground plant matter. We propose that these disturbances may have caused a decrease in biomass accumulation and likely fungal activity. This hypothesis is supported by significant increases in fungal biomass during the subsequent two burial periods for the Alfisol and the Spodosol when more understory begins to grow and develop within each site (Byrd et al., 2000; Lazaruk et al., 2008).

Wallander et al. (2010) noted that mycelium production increases later in the stand’s rotation after canopy closure in Norway spruce (*Picea* abies) forests. Interestingly, similar amounts of biomass were recovered from pre-harvest 2018 samples and post-planting 2022 samples, even though 2018 samples only had a 3-month burial period as opposed to post-planting samples, which had a six-month burial period. Because of the inconsistent burial period from pre-harvest to post-harvest samples, we make two assumptions: (1) the biomass accumulation of fungi was higher in the pre-harvest samples due to increased mycelium production; (2) biomass would have continued to increase in the bag if it had been left in the ground for longer. Hagenbo et al. (2018) reported that increases in burial periods changed the fungal composition of mesh bags, with growing proportions of mycorrhizal fungi increasing with increasing burial periods up to one year. If biomass had continued to increase and mycelium production would have been highest in pre-harvest, it is possible that this experiment’s pre-harvest treatments were dramatically underestimated. Interestingly, post-harvest results from all three burial periods are relatively consistent. The current data shows biomass accumulation in the Alfisol could still increase over time from samples collected in 2020 to 2022, while biomass collected from the Spodosol may have plateaued only one year post-planting in 2021, implying that mycelium production and recovery from disturbances may also be site-dependent. These site-specific patterns may influence P allocation and indicate a need for assessments across many soils to understand mycorrhizal communities (Averill and Hawkes, 2016).

Interestingly, only P-treated mesh bags from pre-harvest 2018 and post-planting 2020 had any biomass response to P carryover treatments. Biomass was lower in plots fertilized with P during the pre-harvest burial period in 2018 but higher in plots receiving higher rates of P carryover in 2020. From these results, we hypothesize a shift in community composition from specialized fungi for P acquisition from the pre-harvest burial period in 2018 to more generalist exploration and exploitation types of fungi post-planting. This assumption is supported by the mesh bags’ community composition, which shows that many saprotrophic groups are present and likely more active due to the increased amounts of organic matter decomposition occurring in the field. There were relationships for some carryover treatments between biomass collected in the mesh bags and the number of taxa extracted from those bags. This positive relationship between the number of species and biomass occurred on the 0 + 0 P Alfisol carryover treatment and the 121 + 0 P Spodosol carryover treatment. This result implies that the number of species in the bags was proportional to increased biomass within the bags under certain circumstances. An odd result implies that the more diverse the community in the bag is, the more biomass is accumulated. This could be a result of increased competing vegetation or organic material decomposition.

Looking at the ECM community in the mesh bags, the responses to site and P carryover treatments indicate that the ECM community is responsive to changes in environment and nutrient availabilities. These changes to the ECM’s abundances and community compositions within the bags suggest that the ECM communities between the two sites are distinct from each other. However, differential abundance analysis of the ECM community between sites found no significantly different genera for either the mesh bags or the rhizospheres. Only a *Denospora* genera was differentially abundant in the 121 + 0 P and 40 + 45 P carryover treatments compared to the 0 + 0 P control treatment. Differential abundance analysis of ECM taxa found no difference between the two soils in the mesh bags or the rhizospheres. Rhizosphere ECM and non-ECM samples were significantly more conserved regarding overall diversity between samples than the mesh bags for the datasets. This reduction in diversity was expected as we sampled closer to the *P. taeda* roots. The microbiome revealed that Spodosol rhizosphere samples had significantly less taxa diversity than the Alfisol for ECM and non-ECM datasets. Many taxa were differentially abundant between the two sites, with members of Ascomycota and Basidiomycota strongly associated with the Spodosols over the Alfisol. This further indicates that the rhizosphere community of ECM inhabiting *P. taeda* in these plots depends on differences in soil texture and nutrient content (Khalid et al., 2022). Only *Chloridium* sp. and *Acephala macrosclerotiorum* were found to be shared between the two sites on the rhizosphere. These two taxa could vary from mutualistic, neutral, or parasitic symbiosis under certain conditions (Münzenberger et al., 2009).

Many of the ECM taxa we found with occurrence on the rhizosphere of the *P. taeda* trees are also known colonizers of *P. taeda* from other studies (Hackman et al., 2022). *Rhizopogon* and *Laccaria* sp. are known to improve the growth and uptake of P in *P. taeda* (Ford et al., 1985; Desai et al., 2014). Differential abundance analysis by carryover treatment on the non-ECM dataset revealed that *Umbelopsis isabellina* was found on the rhizosphere in 121 + 0 P and 40 + 45 P carryover treatments. *Umbelopsis isabelline* belongs to the phylum *Mucoromycota* and is used in the production of biomaterials, specifically chitosan, a deacetylated homopolymer of chitin with various applications in medicine, agriculture, and wastewater industries (Kumar, 2000). Due to these applications, this species has been studied extensively and is highly sensitive to changes in inorganic P (Ye et al., 2015; Dzurendova et al., 2022). Although this species is not known to be ectomycorrhizal, its response to P as a possible biological indicator of P deficiency or surplus on the rhizosphere is an interaction needing further exploration.

## Conclusion

The ECM community and its contributions to plantation pine management have undergone extensive empirical studies in the last few decades. Currently, forest managers think of management in terms of trees alone and do not consider the ECM community as something that can be manipulated or managed. Breaking this dogma is going to require careful expansion into the possibilities and utilizations that this potential new branch of forest management could have. Using the mesh bag capture method, we found that fungal biomass is directly affected by multiple factors, including site, P carryover treatments, mesh bag treatments, and burial periods. Biomass responses to carryover were highly dependent on the burial period and mesh bag treatments. These results support the method’s feasibility to indicate P deficiencies under certain biotic and abiotic conditions. Community composition analysis provides evidence for site-specific ECM taxa, and some non-ECM taxa are associated with *P. taeda* and may be related to increased P uptake. This data further improves our understanding of host-ECM interactions, which can lead to innovative inoculation strategies for P uptake optimization for *P. taeda* in the future. Identifying genera colonizing *P. taeda* and optimizing for gathering P in specific sites and environments will be vital in creating future experimental designs targeting direct applications of ECM with the appropriate conditions to maximize plant productivity.

## Limitations

Rhizosphere sampling was performed by inserting root clippings into a centrifuge tube and vortexing glass beads to remove soil particles from the roots. There is a high probability that fine roots could have been removed from the root clippings, potentially contaminating the rhizosphere samples with mycorrhized fine roots. Although there is a potential for contamination of the rhizosphere samples, we propose that this is still in line with the objectives of the experiment to determine whether similar ECM were present on the roots vs. the mesh bags. Due to harvest operations, our initial plan to have a six-month burial period pre-harvest was cut short to only a three-month burial period. Consistent burial periods should be maintained throughout the experiment if possible.

## Data availability statement

The original contributions presented in the study are publicly available. This data can be found here: NCBI BioProject, PRJNA1111940.

## Ethics statement

The manuscript presents research on animals that do not require ethical approval for their study.

## Author contributions

JH: Conceptualization, Data curation, Formal analysis, Funding acquisition, Investigation, Methodology, Project administration, Software, Validation, Visualization, Writing – original draft, Writing – review & editing. AW: Project administration, Supervision, Writing – review & editing, Methodology. DC: Methodology, Project administration, Supervision, Writing – review & editing. BS: Methodology, Project administration, Supervision, Writing – review & editing. CA: Methodology, Supervision, Writing – review & editing, Conceptualization. RV: Methodology, Supervision, Writing – review & editing, Conceptualization. KG: Methodology, Supervision, Writing – review & editing, Project administration. RC: Supervision, Writing – review & editing, Funding acquisition, Project administration, Resources.

## Supplementary material

Supplementary Figure 1
**(A)** 2018 pre-harvest P-treated mesh bags by fertilization treatment **(B)** 2020 post-planting P-treated mesh bags by fertilization treatment **(C)** 2021 post-planting P-treated mesh bags by P fertilization **(D)** 2022 post-planting P-treated mesh bags by fertilization treatment. Fungal biomass by P fertilization treatment averaged together by site and separated by all four sampling periods. Post-planting, in 2020, fungal biomass in the P-treated bags responded positively to higher P-fertilization rates (p-value = 0.0294), while increasing P rates negatively affected biomass P-treated mesh bags in 2018 (p-value = 0.0130). P fertilization treatments, nor the fertilized treatment (40 + 45 P).

Supplementary Figure 2 Regression of Biomass and Number of Species {calculated via the (Exp [Shannon diversity index(H)]} by P carryover treatment and site. Standard linear regressions were performed on individual treatments to determine if biomass had a relationship to the number of taxa within the mesh Bags. In two P carryover treatments out of 8, the control (0 + 0 P) for the Alfisol **(A)** and the high (121 + 0 P) for the Spodosol **(B)** had relatively strong relationships between the mesh bag number of species and biomass.
