# Supplementary figures and images for: Fungal biomass and ectomycorrhizal community assessment of phosphorus responsive Pinus taeda plantations
Source: Front Fungal Biol. 2024 May 28;5:1401427. doi: 10.3389/ffunb.2024.1401427 (PMC11165416; doi:10.3389/ffunb.2024.1401427)

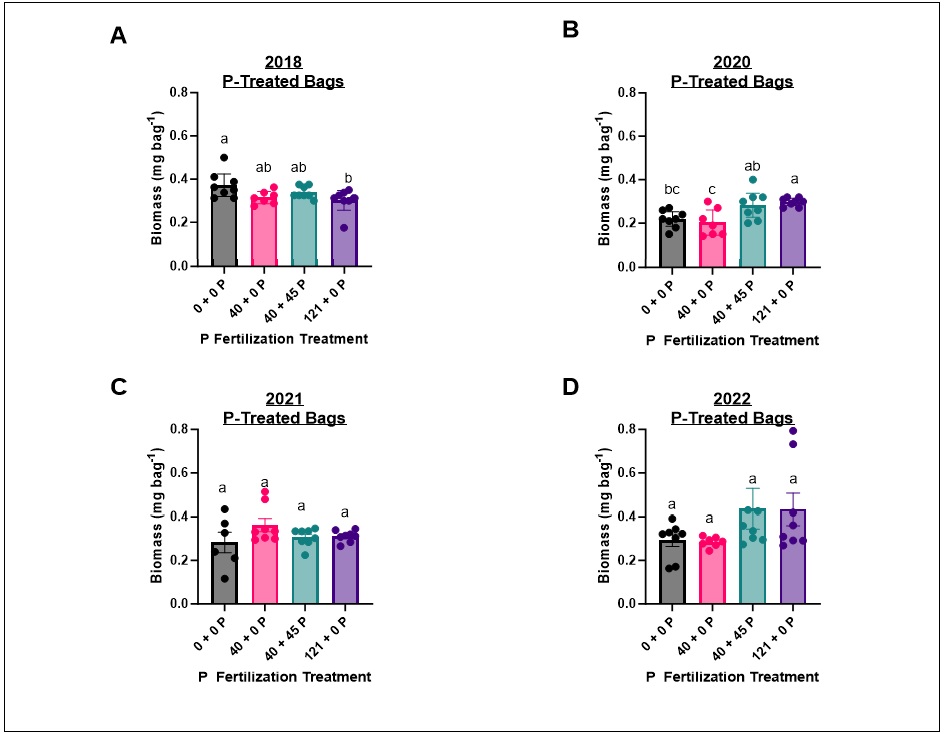

Supplement: Supplementary Figure 1 — (A) 2018 pre-harvest P-treated mesh bags by fertilization treatment (B) 2020 post-planting P-treated mesh bags by fertilization treatment (C) 2021 post-planting P-treated mesh bags by P fertilization (D) 2022 post-planting P-treated mesh bags by fertilization treatment. Fungal biomass by P fertilization treatment averaged together by site and separated by all four sampling periods. Post-planting, in 2020, fungal biomass in the P-treated bags responded positively to higher P-fertilization rates (p-value = 0.0294), while increasing P rates negatively affected biomass P-treated mesh bags in 2018 (p-value = 0.0130). P fertilization treatments, nor the fertilized treatment (40 + 45 P). [file Image_1.jpeg]

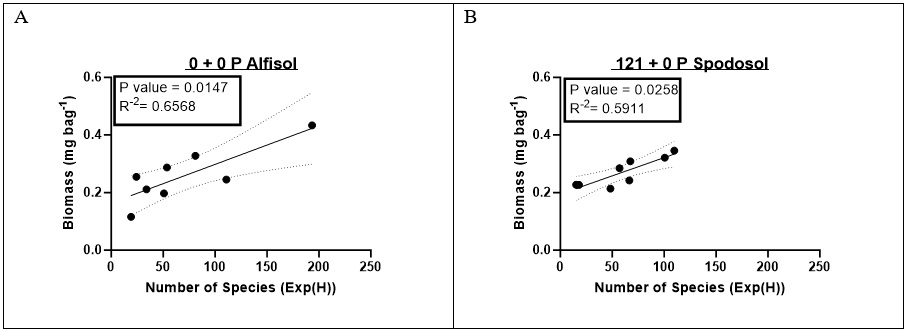

Supplement: Supplementary Figure 2 — Regression of Biomass and Number of Species {calculated via the (Exp [Shannon diversity index(H)]} by P carryover treatment and site. Standard linear regressions were performed on individual treatments to determine if biomass had a relationship to the number of taxa within the mesh Bags. In two P carryover treatments out of 8, the control (0 + 0 P) for the Alfisol (A) and the high (121 + 0 P) for the Spodosol (B) had relatively strong relationships between the mesh bag number of species and biomass. [file Image_2.jpeg]
