# Supplementary material for: Fungal biomass and ectomycorrhizal community assessment of phosphorus responsive Pinus taeda plantations
Source: Front Fungal Biol. 2024 May 28;5:1401427. doi: 10.3389/ffunb.2024.1401427 (PMC11165416; doi:10.3389/ffunb.2024.1401427)
Supplement: Supplementary file 3 [file Table_1.docx]

***Supplementary Table 1:*** *Venn diagram information for mesh bag treatments and rhizosphere.*

| \| **`P-Treated`** \| \| \| **P treated & P untreated & rhizosphere** \| \| \| \| --- \| --- \| --- \| --- \| --- \| --- \| \| ASV \| Genus \| Species \| ASV \| Genus \| Species \| \| ASV160 \| *Russula* \| *andaluciana* \| ASV254 \| *Laccaria* \| *spp.* \| \| ASV1294 \| *Acephala* \| *macrosclerotiorum* \| ASV329 \| *Tomentella* \| *spp.* \| \| ASV4075 \| *Densospora* \| *sp.* \| ASV373 \| *Pisolithus* \| *arhizus* \| \| ASV4622 \| *Entoloma* \| *sp.* \| ASV376 \| *Pisolithus* \| *arhizus* \| \| ASV4695 \| *Entoloma* \| *sp.* \| ASV394 \| *Rhizopogon* \| *spp.* \| \| ASV4961 \| *Densospora* \| *sp.* \| ASV512 \| *Protubera* \| *spp.* \| \| ASV5210 \| *Sistotrema* \| *brinkmannii* \| ASV526 \| *Rhizopogon* \| *spp.* \| \| ASV5330 \| *Amphinema* \| *sp.* \| ASV837 \| *Sistotrema* \| *spp.* \| \| ASV6578 \| *Sistotrema* \| *oblongisporum* \| ASV947 \| *Suillus* \| *cothurtus* \| \| ASV7118 \| *Meiorganum* \| *curtisii* \| ASV1270 \| *Wilcoxina* \| *mikolae* \| \| ASV7442 \| *Retiboletus* \| *visp.ceipes* \| ASV2099 \| *Chloridium* \| *spp.* \| \| ASV7828 \| *Inocybe* \| *subradiata* \| ASV2140 \| *Xerocomus* \| *spp.* \| \| ASV8188 \| *Strobilomyces* \| *confusus* \| **Rhizosphere** \| \| \| \| ASV8635 \| *Sistotrema* \| *diademiferum* \| ASV3584 \| *Pisolithus* \| *arhizus* \| \| ASV8689 \| *Sebacisna* \| *sp.* \| ASV3723 \| *Acephala* \| *macrosclerotiom* \| \| ASV8783 \| *Pisolithus* \| *arhizus* \| ASV4125 \| *Lyophyllum* \| *spp.* \| \| ASV9319 \| *Sphaerosporella* \| *sp.* \| ASV6482 \| *Sphaerosporella* \| *spp.* \| \| ASV9332 \| *Entoloma* \| *sp.* \| ASV7343 \| *Rhizopogon* \| *spp.* \| \| ASV9414 \| *Protubera* \| *sp.* \| ASV9698 \| *Entoloma* \| *infula* \| \| ASV9871 \| *Russula* \| *sp.* \| ASV9703 \| *Tomentella* \| *spp.* \| \| ASV9893 \| *Inocybe* \| *sp.* \| **P-Treated and P Untreated** \| \| \| \| ASV9951 \| *Boletus* \| *pseudopinophilus* \| ASV245 \| *Protubera* \| *spp.* \| \| ASV9952 \| *Acephala* \| *sp.* \| ASV361 \| *Wilcoxispp.* \| *mikolae* \| \| ASV10417 \| *Cortisnarius* \| *sp.* \| ASV977 \| *Sphaerosporella* \| *spp.* \| \| ASV10463 \| *Hebeloma* \| *sp.* \| ASV1372 \| *Protubera* \| *spp.* \| \| ASV10503 \| *Russula* \| *adusta* \| ASV1437 \| *Sphaerosporella* \| *spp.* \| \| ASV10865 \| *Hydnum* \| *sp.* \| ASV1502 \| *Entoloma* \| *scabiosum* \| \| ASV10931 \| *Entoloma* \| *infula* \| ASV1740 \| *Rhizopogon* \| *spp.* \| \| ASV8319 \| *Suillus* \| *cothurnatus* \| ASV2206 \| *Entoloma* \| *spp.* \| \| ASV10498 \| *Clavulina* \| *sp.* \| ASV3543 \| *Entoloma* \| *spp.* \| \| **`P-Untreated`** \| \| \| ASV4890 \| *Entoloma* \| *spp.* \| \| ASV1459 \| *Suillus* \| *cothurnatus* \| ASV5197 \| *Densospora* \| *spp.* \| \| ASV1721 \| *Chloridium* \| *aseptatum* \| ASV5332 \| *Sistotrema* \| *diademiferum* \| \| ASV1758 \| *Pisolithus* \| *arhizus* \| ASV5429 \| *Tomentella* \| *spp.* \| \| ASV2772 \| *Sphaerosporella* \| *sp.* \| ASV6197 \| *Densospora* \| *spp.* \| \| ASV3239 \| *Peziza* \| *ostracoderma* \| ASV6336 \| *Laccaria* \| *longipes* \| \| ASV3680 \| *Suillus* \| *cothurnatus* \| ASV6859 \| *Laccaria* \| *spp.* \| \| ASV4114 \| *Entoloma* \| *luteofuscum* \| ASV7128 \| *Delastria* \| *spp.* \| \| ASV4181 \| *Acephala* \| *macrosclerotiorum* \| ASV9180 \| *Tomentella* \| *spp.* \| \| ASV4377 \| *Tropicoporus* \| *texanus* \| ASV118 \| *Pisolithus* \| *arhizus* \| \| ASV4933 \| *Densospora* \| *sp.* \|  \|  \|  \| \| ASV5148 \| *Acephala* \| *macrosclerotiorum* \|  \|  \|  \| \| ASV5827 \| *Amanita* \| *sp.* \|  \|  \|  \| \| ASV6468 \| *Tulasnella* \| *tomaculum* \|  \|  \|  \| \| ASV6610 \| *Tylopilus* \| *rubrobrunneus* \|  \|  \|  \| \| ASV6957 \| *Xerocomus* \| *illudens* \|  \|  \|  \| \| ASV7614 \| *Lactarius* \| *sp.* \|  \|  \|  \| \| ASV7668 \| *Sphaerosporella* \| *sp.* \|  \|  \|  \| \| ASV7915 \| *Suillus* \| *decipiens* \|  \|  \|  \| \| ASV7934 \| *Cortisnarius* \| *rubeus* \|  \|  \|  \| \| ASV8004 \| *Cortisnarius* \| *sp.* \|  \|  \|  \| \| ASV8484 \| *Entoloma* \| *sp.* \|  \|  \|  \| \| ASV8982 \| *Amanita* \| *morrisii* \|  \|  \|  \| \| ASV9006 \| *Boletus* \| *violaceofuscus* \|  \|  \|  \| \| ASV9367 \| *Sistotrema* \| *coronilla* \|  \|  \|  \| \| ASV9460 \| *Inocybe* \| *paludinella* \|  \|  \|  \| \| ASV9573 \| *Inocybe* \| *petiginosa* \|  \|  \|  \| \| ASV9662 \| *Cortisnarius* \| *sp.* \|  \|  \|  \| \| ASV10096 \| *Retiboletus* \| *vinaceipes* \|  \|  \|  \| \| ASV10267 \| *Piloderma* \| *sp.* \|  \|  \|  \| \| ASV10332 \| *Sistotrema* \| *raduloides* \|  \|  \|  \| \| ASV10446 \| *Tomentella* \| *sp.* \|  \|  \|  \| \| ASV10451 \| *Retiboletus* \| *vinaceipes* \|  \|  \|  \| \| ASV10666 \| *Inocybe* \| *sp.* \|  \|  \|  \| \| ASV10742 \| *Inocybe* \| *subcarpta* \|  \|  \|  \| \| ASV10771 \| *Boletus* \| *sp.* \|  \|  \|  \| \| ASV10905 \| *Tylopilus* \| *griseolivaceus* \|  \|  \|  \| \| ASV1148 \| *Protubera* \| *sp.* \|  \|  \|  \| \| ASV6009 \| *Suillus* \| *decipiens* \|  \|  \|  \| \| ASV9457 \| *Tropicoporus* \| *linteus* \|  \|  \|  \| \| ASV10906 \| *Amphinema* \| *sp.* \|  \|  \|  \| \| ASV10965 \| *Scleroderma* \| *sp.* \|  \|  \|  \| |
| --- | --- | --- | --- | --- | --- | --- | --- | --- | --- | --- | --- | --- | --- | --- | --- | --- | --- | --- | --- | --- | --- | --- | --- | --- | --- | --- | --- | --- | --- | --- | --- | --- | --- | --- | --- | --- | --- | --- | --- | --- | --- | --- | --- | --- | --- | --- | --- | --- | --- | --- | --- | --- | --- | --- | --- | --- | --- | --- | --- | --- | --- | --- | --- | --- | --- | --- | --- | --- | --- | --- | --- | --- | --- | --- | --- | --- | --- | --- | --- | --- | --- | --- | --- | --- | --- | --- | --- | --- | --- | --- | --- | --- | --- | --- | --- | --- | --- | --- | --- | --- | --- | --- | --- | --- | --- | --- | --- | --- | --- | --- | --- | --- | --- | --- | --- | --- | --- | --- | --- | --- | --- | --- | --- | --- | --- | --- | --- | --- | --- | --- | --- | --- | --- | --- | --- | --- | --- | --- | --- | --- | --- | --- | --- | --- | --- | --- | --- | --- | --- | --- | --- | --- | --- | --- | --- | --- | --- | --- | --- | --- | --- | --- | --- | --- | --- | --- | --- | --- | --- | --- | --- | --- | --- | --- | --- | --- | --- | --- | --- | --- | --- | --- | --- | --- | --- | --- | --- | --- | --- | --- | --- | --- | --- | --- | --- | --- | --- | --- | --- | --- | --- | --- | --- | --- | --- | --- | --- | --- | --- | --- | --- | --- | --- | --- | --- | --- | --- | --- | --- | --- | --- | --- | --- | --- | --- | --- | --- | --- | --- | --- | --- | --- | --- | --- | --- | --- | --- | --- | --- | --- | --- | --- | --- | --- | --- | --- | --- | --- | --- | --- | --- | --- | --- | --- | --- | --- | --- | --- | --- | --- | --- | --- | --- | --- | --- | --- | --- | --- | --- | --- | --- | --- | --- | --- | --- | --- | --- | --- | --- | --- | --- | --- | --- | --- | --- | --- | --- | --- | --- | --- | --- | --- | --- | --- | --- | --- | --- | --- | --- | --- | --- | --- | --- | --- | --- | --- | --- | --- | --- | --- | --- | --- | --- | --- | --- | --- | --- | --- | --- | --- | --- | --- | --- | --- | --- | --- | --- | --- | --- | --- | --- | --- | --- | --- | --- | --- | --- | --- | --- | --- | --- | --- | --- | --- | --- | --- | --- | --- | --- | --- | --- | --- | --- | --- | --- | --- | --- | --- | --- | --- | --- | --- | --- | --- | --- | --- | --- | --- | --- | --- | --- | --- | --- | --- | --- | --- | --- | --- | --- | --- | --- | --- | --- | --- | --- | --- | --- | --- | --- | --- | --- | --- | --- | --- | --- | --- | --- | --- | --- | --- | --- | --- | --- | --- | --- | --- | --- | --- | --- | --- | --- | --- | --- | --- | --- | --- | --- | --- | --- | --- | --- | --- | --- | --- | --- | --- | --- | --- | --- | --- | --- | --- | --- | --- | --- | --- | --- | --- | --- | --- | --- | --- | --- | --- |
