# Supplementary material for: Fungal biomass and ectomycorrhizal community assessment of phosphorus responsive Pinus taeda plantations
Source: Front Fungal Biol. 2024 May 28;5:1401427. doi: 10.3389/ffunb.2024.1401427 (PMC11165416; doi:10.3389/ffunb.2024.1401427)
Supplement: Supplementary file 4 [file Table_2.docx]

***Supplementary Table 2:*** *Top differential abundant taxa between sites with base mean > 100. The log2foldchange indicates the difference in fungal sequence abundance in sites. Basemean is an average of normalized counts taken over all samples. A negative log2Fold implies a differentially abundant taxon in the Spodosol, and a positive log2Fold is for the Alfisol. A log2Fold value of 2 implies a 4-fold increase in that group's count of that taxon. lfcSE is the standard error of the log2Fold estimate. padj = Benjamini-Hochberg adjusted p-value.*

| \| **Mesh Bag Non-ECM by Site** \| \| \| \| \| \| \| \| --- \| --- \| --- \| --- \| --- \| --- \| --- \| \| **Phylum** \| **Genus** \| **Species** \| **baseMean** \| **log2Fold** \| **lfcSE** \| **padj** \| \| Basidiomycota \| *Rhodotorula* \| *dairenensis* \| 1290.65 \| 2.91 \| 0.51 \| 0.0000 \| \| Ascomycota \| *Coniochaeta* \| *fodinicola* \| 674.79 \| 4.80 \| 0.64 \| 0.0000 \| \| Basidiomycota \| *Rhodotorula* \| *tortoises* \| 657.25 \| 4.27 \| 0.95 \| 0.0001 \| \| Ascomycota \| *Trichoderma* \| *Sp.* \| 574.16 \| 2.91 \| 0.58 \| 0.0000 \| \| Ascomycota \| *Penicillium* \| *Sp.* \| 524.50 \| 6.98 \| 0.72 \| 0.0000 \| \| Ascomycota \| *Talaromyces* \| *Sp.* \| 517.02 \| 3.34 \| 0.60 \| 0.0000 \| \| Basidiomycota \| *Rhodotorula* \| *toruloides* \| 313.76 \| -2.99 \| 0.87 \| 0.0034 \| \| Ascomycota \| *Coniochaeta* \| *Sp.* \| 277.19 \| 2.33 \| 0.76 \| 0.0100 \| \| Ascomycota \| *Penicillium* \| *Sp.* \| 241.57 \| -28.95 \| 2.95 \| 0.0000 \| \| Ascomycota \| *Trichoderma* \| *Sp.* \| 208.69 \| -3.31 \| 0.64 \| 0.0000 \| \| Ascomycota \| *Trichoderma* \| *Sp.* \| 197.11 \| 1.56 \| 0.63 \| 0.0426 \| \| Ascomycota \| *Tolypocladium* \| *album* \| 172.94 \| -4.02 \| 1.05 \| 0.0009 \| \| Mortierellomycota \| *Mortierella* \| *Sp.* \| 152.77 \| -5.07 \| 0.72 \| 0.0000 \| \| Ascomycota \| *Penicillium* \| *zonatum* \| 137.28 \| 2.21 \| 0.76 \| 0.0152 \| \| Ascomycota \| *Penicillium* \| *Sp.* \| 131.66 \| -11.80 \| 1.60 \| 0.0000 \| \| Mortierellomycota \| *Podila* \| *humilis* \| 129.30 \| -3.79 \| 0.79 \| 0.0000 \| \| Ascomycota \| *Talaromyces* \| *Sp.* \| 120.49 \| 6.76 \| 0.79 \| 0.0000 \| \| Mucoromycota \| *Umbelopsis* \| *autotrophica* \| 116.26 \| 5.83 \| 0.69 \| 0.0000 \| \| Ascomycota \| *Fusarium* \| *Sp.* \| 114.66 \| 3.48 \| 0.87 \| 0.0005 \| \| **Rhizosphere Non-ECM by Site** \| \| \| \| \| \| \| \| Phylum \| Genus \| Species \| baseMean \| log2Fold \| lfcSE \| padj \| \| Ascomycota \| *Penicillium* \| *rolfsii* \| 2024.53 \| -3.14 \| 1.19 \| 0.0379 \| \| Mucoromycota \| *Umbelopsis* \| *longicollis* \| 1251.40 \| 11.37 \| 2.00 \| 0.0000 \| \| Ascomycota \| *Talaromyces* \| *subaurantiacus* \| 895.42 \| -5.12 \| 1.52 \| 0.0055 \| \| Basidiomycota \| *Rhodotorula* \| *pacifica* \| 716.18 \| -6.26 \| 2.38 \| 0.0379 \| \| Ascomycota \| *Penicillium* \| *zonatum* \| 598.77 \| 5.42 \| 1.61 \| 0.0055 \| \| Ascomycota \| *Penicillium* \| *Sp.* \| 491.01 \| 5.27 \| 1.42 \| 0.0021 \| \| Mucoromycota \| *Umbelopsis* \| *angularis* \| 376.35 \| -7.97 \| 1.82 \| 0.0001 \| \| Ascomycota \| *Talaromyces* \| *Sp.* \| 329.77 \| -7.38 \| 1.85 \| 0.0007 \| \| Ascomycota \| *Trichoderma* \| *koningii* \| 304.39 \| -26.79 \| 2.82 \| 0.0000 \| \| Mucoromycota \| *Umbelopsis* \| *NA* \| 295.90 \| -11.35 \| 1.76 \| 0.0000 \| \| Mucoromycota \| *Umbelopsis* \| *Sp.* \| 277.50 \| 11.85 \| 2.29 \| 0.0000 \| \| Mucoromycota \| *Umbelopsis* \| *isabellina* \| 187.15 \| 6.86 \| 2.09 \| 0.0064 \| \| Ascomycota \| *Penicillium* \| *canariense* \| 186.69 \| 6.72 \| 1.86 \| 0.0026 \| \| Ascomycota \| *Talaromyces* \| *Sp.* \| 177.81 \| -10.62 \| 2.06 \| 0.0000 \| \| Basidiomycota \| *Chionosphaera* \| *cuniculicola* \| 115.15 \| 9.00 \| 2.88 \| 0.0100 \| |
| --- | --- | --- | --- | --- | --- | --- | --- | --- | --- | --- | --- | --- | --- | --- | --- | --- | --- | --- | --- | --- | --- | --- | --- | --- | --- | --- | --- | --- | --- | --- | --- | --- | --- | --- | --- | --- | --- | --- | --- | --- | --- | --- | --- | --- | --- | --- | --- | --- | --- | --- | --- | --- | --- | --- | --- | --- | --- | --- | --- | --- | --- | --- | --- | --- | --- | --- | --- | --- | --- | --- | --- | --- | --- | --- | --- | --- | --- | --- | --- | --- | --- | --- | --- | --- | --- | --- | --- | --- | --- | --- | --- | --- | --- | --- | --- | --- | --- | --- | --- | --- | --- | --- | --- | --- | --- | --- | --- | --- | --- | --- | --- | --- | --- | --- | --- | --- | --- | --- | --- | --- | --- | --- | --- | --- | --- | --- | --- | --- | --- | --- | --- | --- | --- | --- | --- | --- | --- | --- | --- | --- | --- | --- | --- | --- | --- | --- | --- | --- | --- | --- | --- | --- | --- | --- | --- | --- | --- | --- | --- | --- | --- | --- | --- | --- | --- | --- | --- | --- | --- | --- | --- | --- | --- | --- | --- | --- | --- | --- | --- | --- | --- | --- | --- | --- | --- | --- | --- | --- | --- | --- | --- | --- | --- | --- | --- | --- | --- | --- | --- | --- | --- | --- | --- | --- | --- | --- | --- | --- | --- | --- | --- | --- | --- | --- | --- | --- | --- | --- | --- | --- | --- | --- | --- | --- | --- | --- | --- | --- | --- | --- | --- | --- | --- | --- | --- | --- | --- | --- | --- | --- | --- | --- | --- | --- | --- | --- | --- | --- | --- | --- | --- | --- | --- | --- | --- | --- | --- | --- | --- | --- | --- | --- | --- | --- | --- | --- |
